# Prognostic value of neutrophil to lymphocyte ratio in patients with aortic dissection: a systematic review and meta-analysis

**DOI:** 10.3389/fcvm.2025.1631314

**Published:** 2026-01-08

**Authors:** Lilong Li, Cheng Wen, Linyang Xie, Huaping Wu

**Affiliations:** 1 School of Clinical Medicine, North Sichuan Medical College, Nanchong, Sichuan, China; 2 Department of Vascular Surgery, Dazhou Central Hospital, Dazhou, Sichuan, China; 3 School of Medical and Life Sciences, Chengdu University of Traditional Chinese Medicine, Chengdu, Sichuan, China

**Keywords:** aortic dissection, inflammation, meta-analysis, mortality, neutrophil-to-lymphocyte ratio

## Abstract

**Background:**

Aortic dissection (AD) is a highly fatal cardiovascular condition, with mortality exceeding 50% within the first 24 h and postoperative mortality approaching 30%. Inflammatory mechanisms have been implicated in AD pathogenesis, but the prognostic value of the neutrophil-to-lymphocyte ratio (NLR) remains uncertain. This study aimed to synthesize available evidence on NLR as a predictor of adverse outcomes in AD and to inform hypotheses for future research.

**Methods:**

Following PRISMA 2020 guidance, a systematic search was conducted in Web of Science, PubMed, Embase, and the Cochrane Library through December 2024. Study selection, data extraction, and quality assessment were performed independently by two reviewers. Pooled odds ratios (ORs) and 95% confidence intervals (CIs) were estimated using a random-effects model. Heterogeneity and potential bias were evaluated via subgroup analyses, sensitivity testing, and Egger's regression.

**Results:**

Eleven studies (12 comparative groups) comprising 1,792 patients were included (one publication reported two independent cohorts). Eelevated NLR was associated with increased mortality risk (pooled OR = 1.16, 95% CI: 1.05–1.27, *P* = 0.002). This association was significant in cohort studies (*P* = 0.0005) but not in case-control studies (*P* = 0.28). The result remained robust in leave-one-out sensitivity analyses. Substantial publication bias was detected (Egger's *P* < 0.0001). In patients with uncomplicated type B AD managed by endovascular intervention, elevated NLR predicted adverse events within two years (hazard ratio = 1.98, *P* = 0.015).

**Conclusion:**

The evidence indicates that elevated NLR may serve as a potential independent predictor of mortality in AD, supporting its incorporation into risk stratification frameworks. Notwithstanding substantial publication bias—predominantly among smaller studies—sensitivity analyses confirmed the overall result stability. The observed prognostic value aligns with NLR's role in other vascular diseases, consistent with its interpretation as a marker of inflammatory burden. Generalizability is limited by the predominance of small Asian cohorts; therefore, multicenter prospective studies are required to validate these findings and to establish optimal NLR thresholds for individualized management.

**Systematic Review Registration:**

https://www.crd.york.ac.uk/prospero/display_record.php?RecordID=648318, PROSPERO CRD42025648318.

## 1 Introduction

Aortic dissection (AD) results from the interplay between structural abnormalities of the aortic media and hemodynamic disturbances. When the aortic intima is disrupted, hemodynamic forces drive blood into the medial layer, separating the intima and media. The Stanford classification system categorizes these events based on anatomical involvement: Type A aortic dissections (TAAD) affect the ascending aorta, while Type B aortic dissections (TABD) involve the descending thoracic aorta and distal segments, sparing the ascending aorta (1). Epidemiological data indicate an incidence of 2.8–6.0 cases per 100,000 population per year (2). TAAD represents the most common and life-threatening variant of AD. Its mortality exceeds 50% within 24 h of onset and increases by 1%–2% each subsequent hour. Despite surgical intervention, postoperative mortality remains 10%–30% (3, 4). Therefore, prompt and accurate diagnosis and appropriate treatment are crucial.

Inflammatory responses significantly contribute to AD pathogenesis (5). Histopathological studies show macrophage infiltration and increased matrix metalloproteinase (MMP) activity in the aortic wall of AD patients, with MMP-12 identified as a marker of aortic wall disease irrespective of genetic predisposition (6). The neutrophil-to-lymphocyte ratio (NLR) is an accessible marker of systemic inflammation and may predict disease severity and outcomes in cardiovascular conditions (7). Ustaalioglu et al. reported a strong association between elevated NLR and mortality in AD (8), whereas Donmez et al. found higher NLR in both deceased and surviving patients without statistical significance (9). Hung et al.'s 2021 meta-analysis of four TAAD studies identified preoperative NLR as a predictor of in-hospital mortality after surgury (10).

Based on these findings, a meta-analysis evaluating the prognostic significance of NLR in AD patients was conducted. This study aims to synthesize recent evidence to assess NLR's prognostic value, improve risk stratification, and inform individualized intervention strategies, and to propose perspectives on translating inflammatory mechanisms into clinical application for AD patients.

## 2 Materials and methods

### 2.1 Literature search

The systematic review was conducted in accordance with PRISMA 2020 and was prospectively registered in PROSPERO (CRD42025648318). The search methodology, including relevant MeSH terms and keywords, was developed by two independent investigators (LLL and WC). A comprehensive search was performed in PubMed, Embase, Web of Science, and the Cochrane Library from inception through December 2024 using terms including “Aortic Dissection,” “Dissecting Aneurysm,” “dissection of aorta,” “Aortic Dissecting Aneurysm,” “Lymphocyte,” “Neutrophil,” and “neutrophil to lymphocyte ratio (NLR).” The literature search strategy is provided in Supplementary Table S1.

### 2.2 Study selection

Eligibility was assessed according to predefined inclusion criteria: (1) Confirmed diagnosis of AD via computed tomography angiography (CTA); (2) Research examining NLR's prognostic significance in AD; (3) Availability of OR with 95% CI, either directly reported or calculable from provided information; (4) Classification of participants into distinct groups based on NLR levels (high vs. low) using specified cut-off values; (5) Publication of the full article.

Exclusion criteria were: (1) Reviews, comments, meeting abstracts, case reports, and letters; (2) Publications with insufficient statistical data for OR and 95% CI; (3) Studies lacking survival outcome measures; (4) Studies with redundant or overlapping participant cohorts.

Screening and selection were performed independently by two authors (LLL and WC) through review of titles, abstracts, and full texts. Discrepancies were resolved by discussion. One included study (11) contributed two independent comparative groups; therefore, the final analysis included 11 publications providing data for 12 comparative groups.

### 2.3 Data extraction

Data extraction was performed independently by two investigators (LLL and WC), and any disagreements were resolved by consensus. The following variables were extracted: publication year, primary author identification, sample size, research design, median age, geographical location, investigation timeframe, follow-up duration, NLR threshold values, and statistical measures (OR with 95% CIs) pertaining to mortality outcomes in AD.

### 2.4 Quality assessment

The methodological rigor of the included investigations was evaluated using the Newcastle-Ottawa Quality Assessment Scale (NOS). This validated instrument assesses selection, group comparability, and outcome measurement, with a maximum score of nine (12). Studies scoring 7–9 were considered high-quality.

### 2.5 Statistical analysis

Aggregate ORs with 95% CIs were calculated to assess the predictive value of NLR for aortic dissection outcomes. The odds ratio (OR) was the primary metric because it was the most consistently reported effect measure across studies reporting binary outcomes (e.g., deceased vs. survived). Although follow-up durations varied, pooling by OR is an acceptable approach when hazard ratios (HRs) are unavailable, providing a standardized assessment of the association between NLR and mortality odds. Inter-study heterogeneity was quantified utilizing the I^2^ statistic and Cochran's *Q* test (13). When I^2^ exceeded 50%, a random-effects model was adopted; otherwise, a fixed-effects model was applied. Subgroup analyses and sensitivity tests were conducted to assess result consistency and explore heterogeneity sources. Publication bias was assessed by visual inspection of funnel plots and Egger's regression test. Statistical significance was defined as *p* < 0.05. All statistical analyses were performed using Review Manager (version 5.4) and STATA (version 17.0).

## 3 Results

### 3.1 Study characteristics

**Figure 1:**
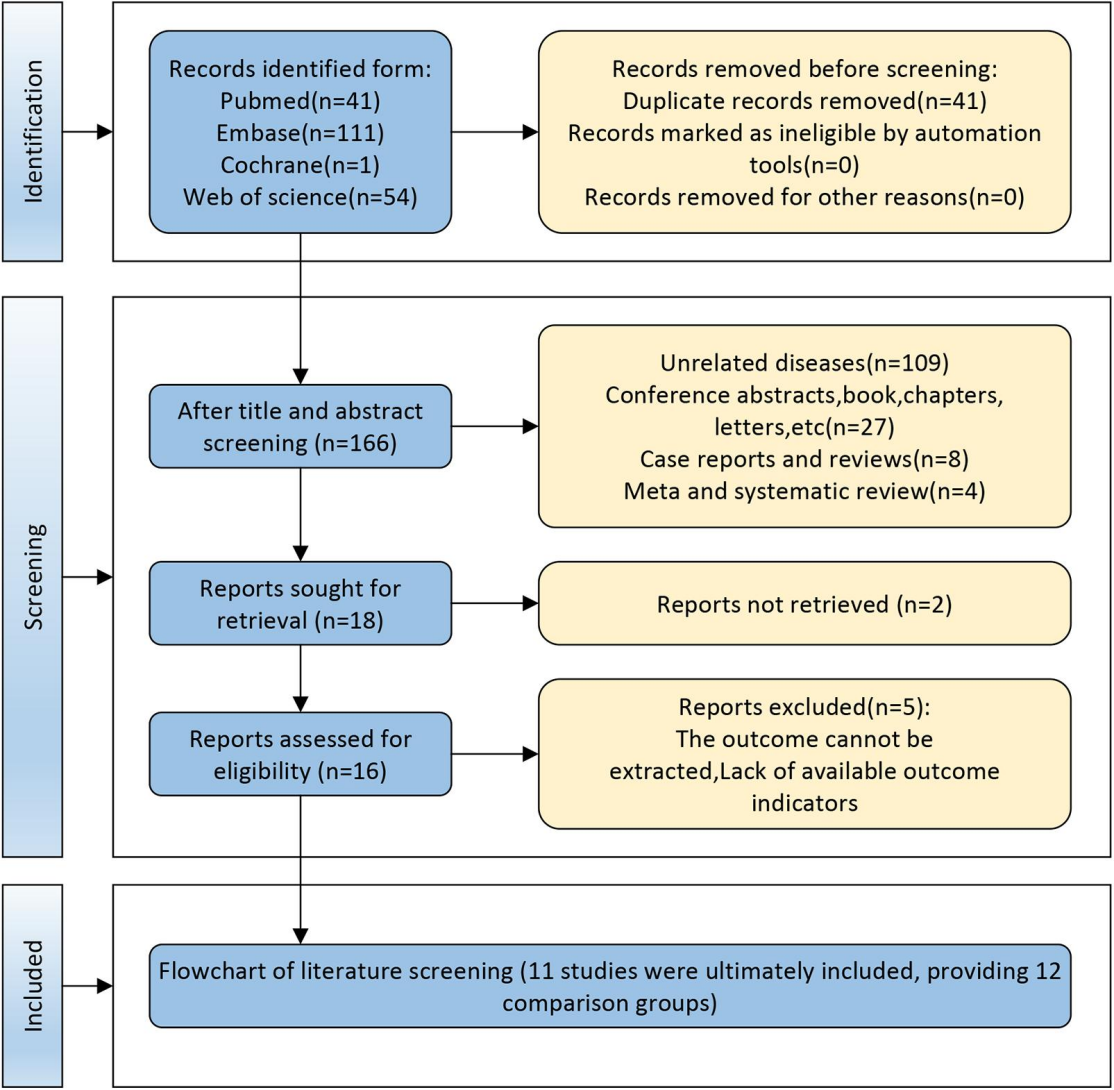
Flow chart of literature screening.

A total of 207 citations were retrieved from database searches. After removal of duplicates (*n* = 41), 148 records were excluded following title and abstract review. Full-text review of 18 studies led to exclusion of seven due to extractable data were insufficient for analysis. The final analysis included 11 studies (12 comparative groups) representing 1,792 patients (8, 11, 14–22) (Figure 1). Zhang et al. (2021) (11) reported two independent cohorts, yielding the 12 comparative groups included in the meta-analysis.

Among the selected studies, seven originated from Turkey and four from China. One publication (11) contained two independent cohorts, yielding eight cohorts and four case-control studies in total. Of the eight cohort analyses, seven were retrospective (8, 11, 14, 15, 21, 22), and one was prospective (16). All studies were published in English, with publication years ranging from 2014 to 2024.

AD cases in all included studies were classified based on Stanford criteria, and patients were categorized into low- and high-NLR groups. Preoperative NLR values were evaluated to assess their prognostic significance for AD. Notably, one study also examined NLR's utility for early differentiation of aortic coarctation. Table 1 summarizes the key characteristics of the included studies.

**Table 1:** Basic characteristics of the included literature.

Author and Year	study period	region	study design	Types of aortic dissection	Number of patients	Gender		Age	NLR cut-off	Quality score
						Male	Female			
Zhang 2021 a	2018–2020	China	cohort study	type A and type B	NA	NA	NA	NA	11.08	7
Zhang 2021 b	2018–2020	China	cohort study	type A and type B	NA	NA	NA	NA	20.22	7
Lafci 2014	2007–2012	Turkey	Case-control study	type A	104	76	28	55.3	NA	8
Oz 2017	NA	Turkey	cohort study	type A	57	9	48	56	NA	7
Ustaalioglu 2024	2018–2023	Turkey	cohort study	type A and type B	103	78	25	73.9	NA	8
Onuk 2015	2004–2014	Turkey	cohort study	type A and type B	200	133	67	58	NA	7
Zhang 2021	2017–2020	China	cohort study	type A	224	172	52	53.2	NA	7
Erdolu 2020	2010–2018	Turkey	Case-control study	type A	118	89	29	57	NA	7
Bedel 2019	2013–2018	Turkey	Case-control study	type A	96	78	18	63.7	NA	7
Yang 2021	2010–2017	China	cohort study	type B	348	298	50	53.7	4.1	8
Zhao 2023	2018–2020	China	cohort study	type A	193	146	47	54.4	NA	8
Kalkan 2017	2009–2013	Turkey	Case-control study	type A	184	134	50	53.1	6	7

### 3.2 Quality assessment of included studies

Using the NOS scale, scores of 7–8 were assigned to all eleven studies, reflecting high methodological quality (Supplementary Tables S2 and S3).

### 3.3 Association between NLR and mortality in aortic dissection

**Figure 2:**
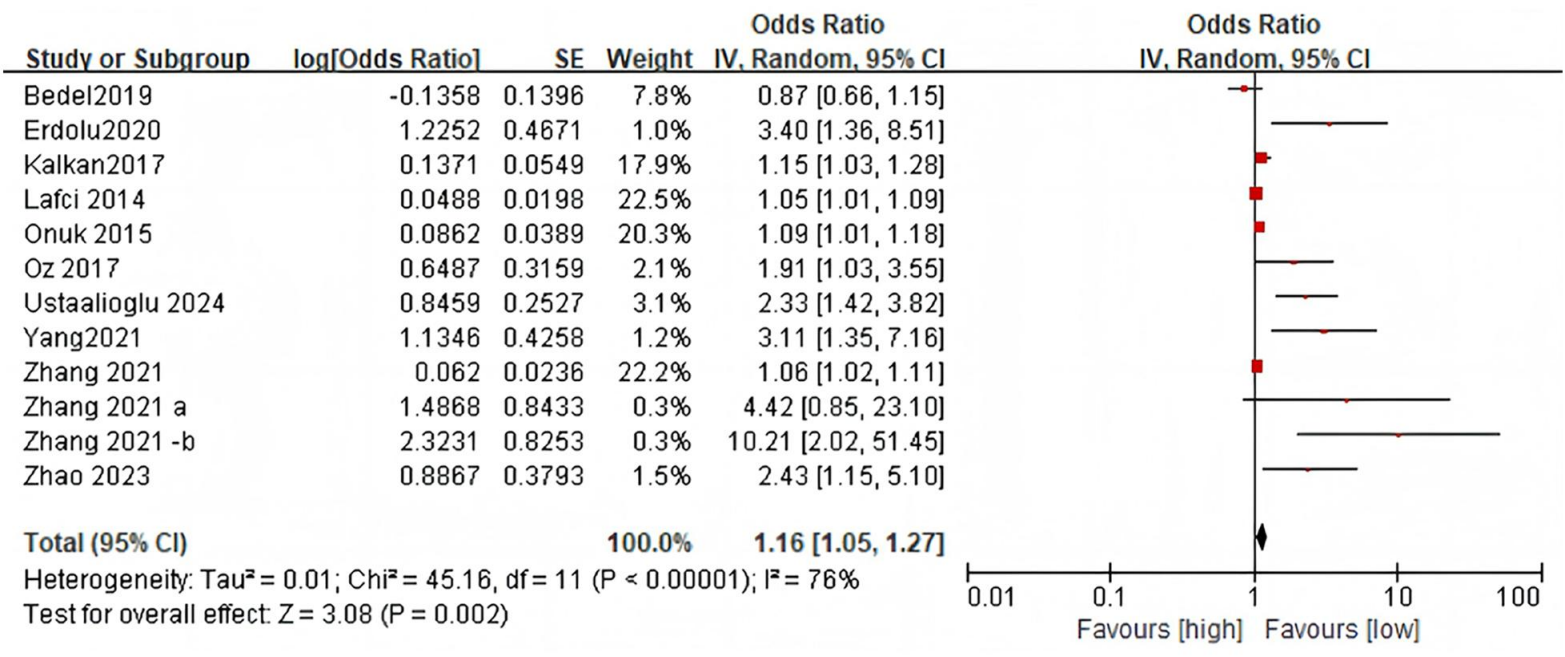
Forest plots for the association between NLR and mortality in patients with AD.

Eleven studies (12 comparative groups) were included, encompassing 1,792 patients; preoperative NLR and its association with clinical outcomes in AD were evaluated in all studies. Notable heterogeneity was observed (I^2^ = 76%, *p* < 0.00001), necessitating use of a random-effects model. The pooled results indicated that elevated preoperative NLR was associated with increased mortality in AD patients (OR = 1.16, 95% CI: 1.05–1.27; *p* = 0.002, Figure 2).

Analyses were stratified by study design, geographic region, sample size, aortic dissection type, and median participant age (Table 2). The predictive significance of NLR for mortality was not observed in the case-control subgroup or in studies with sample sizes ≥ 200. However, significant association of NLR with mortality were retained in other subgroups.

**Table 2:** Pooled OR for NLR in subgroup analyses.

Subgroup	Mortality			
	Study	OR [95%CI]	*P*-value	I^2^
Total	12	1.16 [1.05–1.27]	0.002	76%
Study design				
Cohort	8	1.40 [1.16–1.70]	0.0005	79%
Case-control	4	1.08 [0.94–1.24]	0.28	71%
Sample size				
≥200	3	1.09 [0.99–1.21]	0.09	69%
<200	7	1.27 [1.06–1.53]	0.01	79%
Region				
China	5	2.63 [1.19–5.58]	0.02	81%
Turkey	7	1.14 [1.02–1.27]	0.02	75%
Types of aortic dissection				
A	7	1.09 [1.01–1.19]	0.03	68%
B	1	3.11 [1.35–7.16]	0.008	NA
A and B	4	2.38 [1.08–5.28]	0.003	83%
Mean/median age				
≥55	6	1.15 [1–1.33]	0.05	77%
<55	4	1.20 [1.00–1.43]	0.05	76%

OR, odds ratio; CI, confidence interval; NA, not available.

### 3.4 NLR as a predictor of early adverse outcomes in aortic dissection

One retrospective investigation specifically addressed the prognostic value of NLR for predicting early complications in patients with uncomplicated type B aortic dissection (uTBAD) treated with TEVAR, and therefore was excluded from the meta-analysis (23). 216 individuals diagnosed with uTBAD were followed from January 2015 to December 2018, with a median follow-up of 21 months (IQR: 15–33 months). Early complications were defined as events within two years post-procedure and included overall mortality, types I/II endoleak, stent-graft-induced new entry (SINE), retrograde aortic dissection, severe stroke, paraplegia, and aortic rupture. Twenty-four patients (11.1%) experienced at least one early adverse event: one fatal stroke, thirteen type I/II endoleaks, five retrograde dissections, four recurrent events, and one SINE. The two-year event-free survival rate was 82.7% in patients with elevated NLR values, significantly lower than the 94.6% rate in the low-NLR group. Multivariable regression revealed that higher preoperative NLR independently correlated with an increased risk of two-year adverse outcomes (hazard ratio per SD increment: 1.98; 95% CI: 1.14–3.44; *P* = 0.015). These findings indicate that increased pre-procedural NLR is an independent prognostic marker for early complications after TEVAR in uTBAD.

### 3.5 Sensitivity analysis

**Figure 3:**
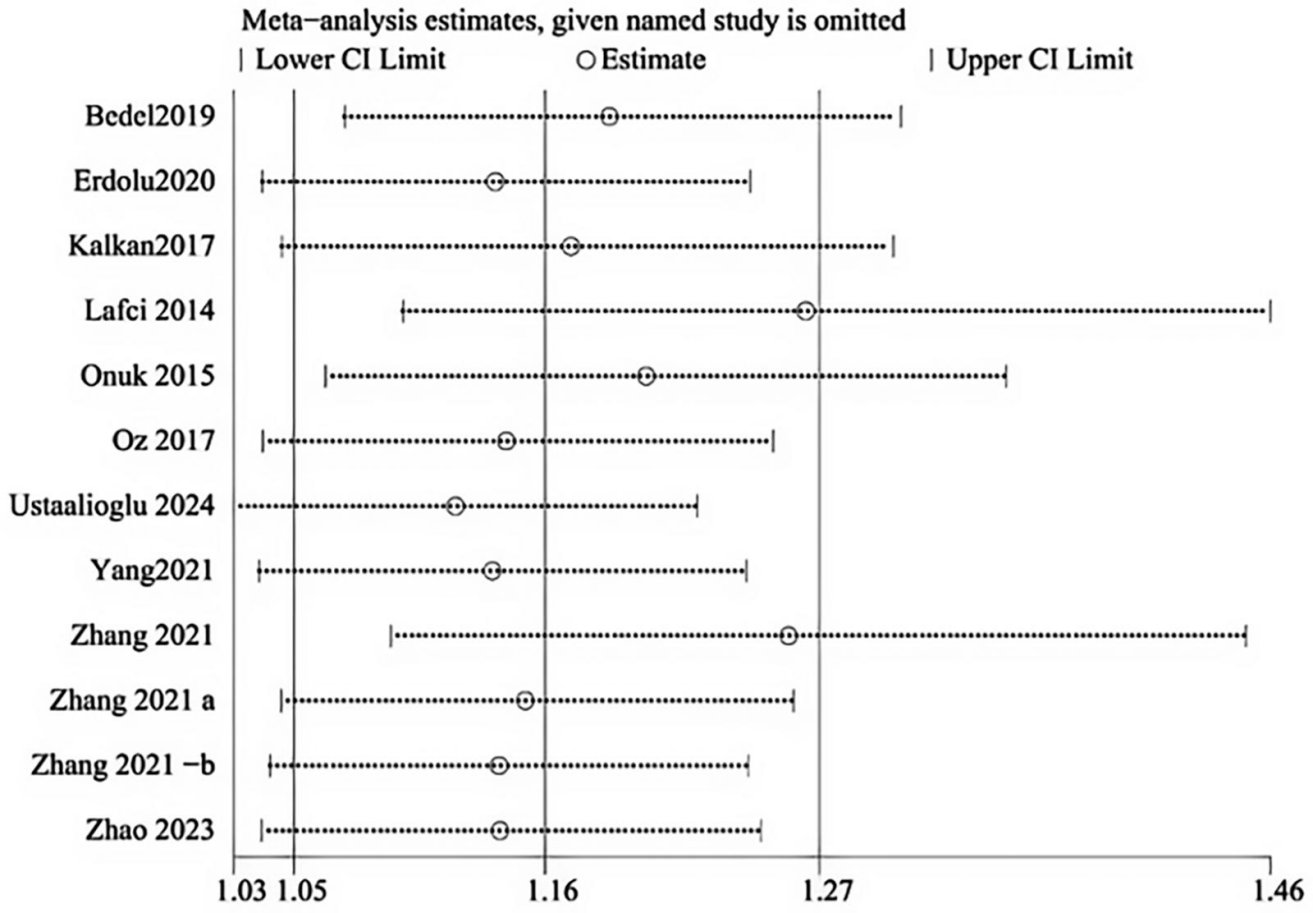
Sensitivity analysis between NLR and mortality in patients with AD.

Leave-one-out sensitivity analysis was performed by systematically excluding each study from the pooled analysis. No single study was found to disproportionately influenced the overall results, supporting the robustness of the meta-analysis (Figure 3).

### 3.6 Publication bias

**Figure 4:**
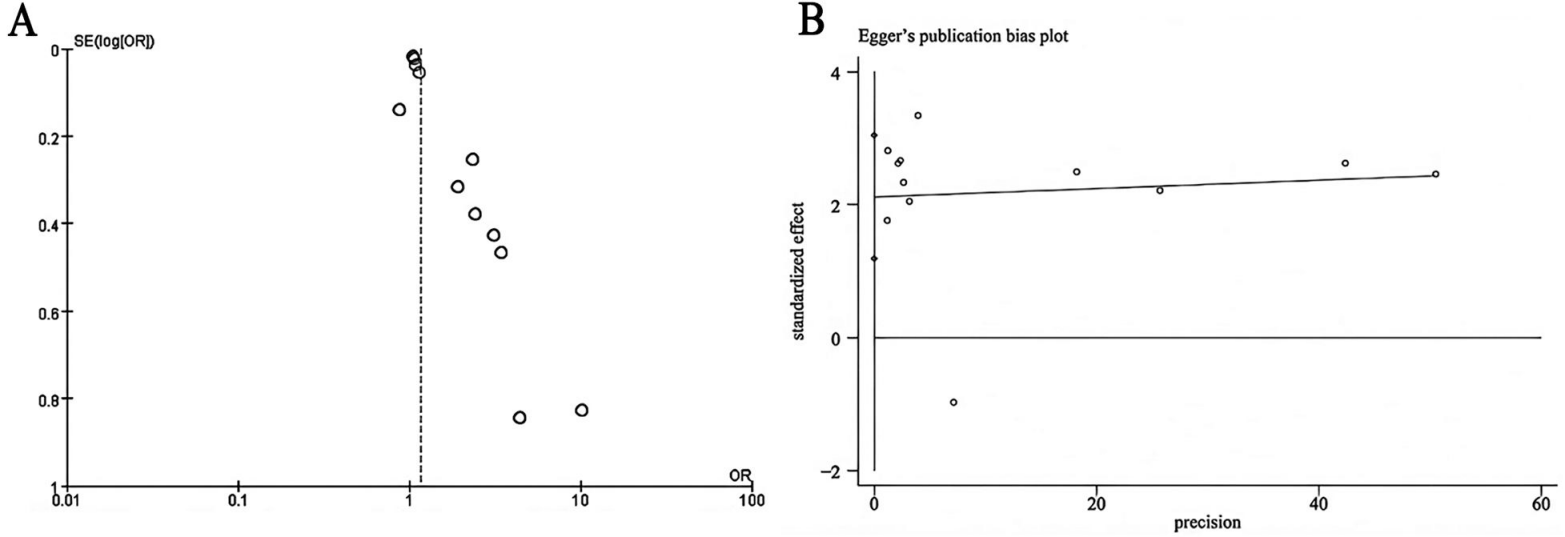
**(A)** funnel plot for the evaluation of publication bias for mortality. **(B)** Egger's test for assessing mortality publication bias.

Visual inspection of the funnel plot revealed notable asymmetry in mortality effect estimates (Figure 4A). Egger's regression test confirmed significant publication bias for mortality outcomes (*p* < 0.0001; Figure 4B). The asymmetry was more pronounced among smaller studies, which also demonstrated stronger effect estimates, raising the possibility of overestimation in these cohorts.

## 4 Discussion

Substantial evidence indicates that AD is closely related to inflammation (24). Although AD is regarded as a multifactorial disease, inflammatory mechanisms is associated with aortic medial degeneration (25). Histopathological examination has revealed marked macrophage and T lymphocyte infiltration and increased expression of apoptosis-related protein. Cifani et al. further elucidated that macrophage recruitment and activation in the aortic wall are early events in Stanford-A AAD; macrophages release proinflammatory cytokines and MMPs that promote matrix degradation and neoangiogenesis (6). Serum inflammatory markers levels may reflect disease severity. Beyond tissue-level inflammation, elevated circulating inflammatory markers are independent predictors of various cardiovascular conditions (26).

The prognostic utility of NLR extends beyond aortic dissection to other acute and chronic vascular conditions. Taurino et al. demonstrated that a preoperative NLR >5 was a strong independent predictor of 30-day amputation (OR: 9.65) and mortality (OR: 9.88) in patients with acute limb ischemia (27). Similarly, Sirignano et al. recently reported that an elevated NLR (>5) was associated with worse long-term limb salvage rates following popliteal artery aneurysm repair, with multivariable analysis identifying NLR as an independent predictor of freedom from major amputation (28). These consistent findings across vascular pathologies reinforce NLR's role as a marker of systemic inflammatory burden in vascular diseases.

Our investigation, by synthesizing available evidence, suggests that elevated NLRs may be associated with heightened mortality risk following surgical intervention in AD patients. Sensitivity analysis confirmed the result's stability, indicating it was not dependent on any single study. However, the observed publication bias may undermine the reliability of our findings. This bias might stem from various factors including geographic region, sample size, and demographic characteristics, necessitating additional investigation for confirmation. A meta-analysis by Chung et al. (10), encompassing four studies and 502 TAAD patients, found that NLR was substantially increased both in those with TAAD and especially in patients who succumbed after surgery. Their findings supported the use of NLR as a practical prognostic tool for in-hospital mortality following TAAD surgery. Recent literature further supports the prognostic capacity of NLR for mortality risk across both TAAD and TBAD (8, 11), which is similar to our results. Therefore, NLR should be monitored for clinical risk stratification and early identification of high-risk patients to permit timely intervention.

Publication bias was evident in our analysis. To address this limitation, subgroup analyses were performed to examine the association between elevated NLR and mortality in AD. The subgroup results showed a significant association in cohort studies (*P* = 0.0005), whereas no significant relationship was found in case-control studies (*P* = 0.28). Cohort studies generally provide higher-quality evidence than case-control studies because exposures are measured before outcomes, establishing temporality and facilitating control of confounding; incidence and other measures can also be directly estimated. In contrast, case-control studies are retrospective and are more susceptible to recall and selection biases and cannot directly calculate incidence. When results conflict, evidence from cohort studies should therefore be prioritized. Analyses of large samples were non-significant results while smaller studies showed significant association, suggesting possible small-study effects. Only three included studies had sample sizes ≥ 200, which may have produced weight imbalance and increased heterogeneity. Therefore, future research incorporating larger cohorts will be essential to confirm NLR's predictive utility.

A methodological distinction was revealed by our subgroup analysis: the association between elevated NLR and mortality was significant in cohort studies (*P* = 0.0005) but not in case-control studies (*P* = 0.28). This discrepancy is plausible and reflects the higher evidentiary strength of cohort designs in prognostic research. Cohort studies, through prospective or longitudinal follow-up, establish temporal sequence—exposure (high NLR) precedes outcome (mortality)—which is fundamental for causal inference and are less susceptible to selection and recall biases than retrospective case-control designs. In case-control studies, in which patients are selected by outcome and prior NLR is assessed, NLR measurement may be affected by post-outcome conditions or biased control selection. Therefore, cohort evidence should be weighted more heavily. Our conclusion that NLR predicts mortality in AD is supported by the more methodologically rigorous cohort studies included in this meta-analysis.

Inflammation processes are central to the development and progression of acute cardiovascular events. Neutrophils, in particular, function as primary mediators in vascular injury and subsequent inflammatory cascades. They rapidly migrate to sites of tissue damage, establishing the initial defensive barrier in inflammatory responses (29). They remove pathogens and cellular debris via phagocytosis and by releasing proteolytic enzymes and reactive oxygen species. When excessive, these mediators cause collateral tissue injury, worsening disease severity and contributing to AD complications (30). This is consistent with findings that neutrophil-derived MMP-9 can trigger acute aortic dissection (6). This functional duality of neutrophils holds considerable significance in understanding AD pathophysiology. Elevated neutrophil counts in AD patients suggest active inflammatory involvement and may correlate with disease severity. Consequently, the NLR emerges as an effective parameter reflecting inflammatory burden.

Lymphocytes are essential components of adaptive immunity, providing protection against viral and bacterial pathogens (31). During severe vascular events such as AD, lymphocytes contribute to immune regulation and tissue repair. Lymphocytes undergo antigen-specific proliferation, generating specialized immune responses that modulate inflammation. T lymphocytes exert anti-inflammatory and tissue-protective effects, while B lymphocytes produce antibodies that neutralize pathogens (32). Lymphocyte functionality significantly influences AD progression and outcomes. Lymphopenia typically indicates immunosuppression in severe infection or systemic inflammation. Thus, elevated NLR, reflecting neutrophilia and lymphopenia, provides an objective measure of inflammatory severity and tissue stress. Increased NLR in AD patients indicates this immunological imbalance and is associated with higher mortality, supporting NLR's potential utility in clinical risk stratification, which warrants future investigation.

Significant publication bias was identified, particularly among smaller studies, which also showed larger effect estimates. This pattern suggests that the pooled effect might be inflated due to preferential publication of positive findings from underpowered studies. However, several factors support the robustness of our primary conclusion. First, the leave-one-out sensitivity analysis confirmed that no single study disproportionately influenced the pooled estimate. Second, the significant association persisted in subgroup analyses of cohort studies—a design generally less susceptible to recall and selection biases. Third, the biological plausibility of NLR as a marker of systemic inflammation is well-established in cardiovascular pathophysiology. Nevertheless, the influence of small-study effects cannot be fully excluded, and the observed OR should be interpreted as potentially representing the upper bound of the true effect size. Future well-powered prospective studies are needed to provide a more precise estimate.

Several limitations of this meta-analysis should be noted. First, all included investigations were conducted in Asia, predominantly Turkey and China, limiting generalizability to other regions. Second, most studies had small sample sizes, with limited large-scale experimental data. Third, substantial heterogeneity in NLR cut-off values (ranging from 4.1 to 20.22) was observed, which hinders direct clinical application of a specific NLR value. An exploratory subgroup analysis by cut-off value could not be meaningfully performed because only 4 out of 12 comparative groups reported specific thresholds. Without individual patient data, harmonization of NLR values or derivation of a unified cut-off was not feasible. Additionally, publication bias was evident and may have favored studies with significant findings or those aligning with prevailing hypotheses, thus failing to comprehensively represent the research landscape. Future investigations require expanded datasets to enhance robustness and address heterogeneity in these findings.

Furthermore, it is crucial to recognize the potential impact of regional variations in clinical practice patterns on AD management. Our analysis included studies conducted only in Turkey and China, and differences in healthcare systems, surgical expertise, timing of intervention, postoperative care protocols, and availability of advanced therapies between these and other regions may influence patient outcomes independently of the NLR. While NLR, as a measure of systemic inflammation, is likely to retain its fundamental prognostic value across settings, the specific risk thresholds and the magnitude of its effect could be modulated by these external factors. For instance, a healthcare setting with rapid access to specialized care might mitigate the risk associated with a high NLR, thereby altering its predictive performance. Therefore, the generalizability of our findings to populations in Europe, North America, or other regions should be approached with caution. Future research should not only validate NLR's utility in multi-ethnic cohorts but also seek to understand how it integrates into specific clinical pathways and healthcare contexts to ensure its effective implementation.

## 5 Conclusion

Our results suggest an association between higher NLR and elevated mortality risk in individuals with aortic dissection, with both subgroup and sensitivity analyses supporting the robustness of this relationship. Despite the observed publication bias and potential overestimation in smaller studies, the consistency of NLR's prognostic value across multiple study designs and its biological plausibility strengthen its candidacy as a useful clinical biomarker. The consistency of NLR's prognostic value across aortic dissection, acute limb ischemia, and popliteal artery aneurysm repair underscores its utility as a general inflammatory biomarker in vascular diseases. However, considering this study's limitations—including restricted sample sizes and predominant focus on Asian populations—additional international, multicenter prospective clinical trials are warranted to further validate the relationship between NLR and AD mortality, to determine optimal patient populations, and to establish precise parameters for clinical implementation.

## Data Availability

The original contributions presented in the study are included in the article/Supplementary Material, further inquiries can be directed to the corresponding author.

## References

[1] Sherk WM Khaja MS Williams DM. Anatomy, pathology, and classification of aortic dissection. Tech Vasc Interv Radiol. (2021) 24(2):100746. 10.1016/j.tvir.2021.100746 34602269

[2] Surgeons CoGVoCAoC. Chinese Experts’ consensus of standardized diagnosis and treatment for acute aortic syndrome(2021). Chin J Thorac Cardiovasc Surg. (2021) 37(5):257–69. 10.3760/cma.j.cn112434-20210319-00103

[3] Evangelista A Isselbacher EM Bossone E Gleason TG Eusanio MD Sechtem U Insights from the international registry of acute aortic dissection: a 20-year experience of collaborative clinical research. Circulation. (2018) 137(17):1846–60. 10.1161/CIRCULATIONAHA.117.031264 29685932

[4] Junming Zhu SC. Treatment of acute type A aortic dissection: towards doing better. Chin J Vasc Surg. (2021) 06(1):1–4. 10.3760/cma.j.cn101411-20210201-00013

[5] Zhang Han LY Zhang J. Molecular biological mechanism of acute aortic dissection. Chin J Vasc Surg. (2023) 08(2):226–31. 10.3760/cma.j.cn101411-20221128-00106

[6] Cifani N Proietta M Tritapepe L Di Gioia C Ferri L Taurino M Stanford-A acute aortic dissection, inflammation, and metalloproteinases: a review. Ann Med. (2015) 47(6):441–6. 10.3109/07853890.2015.1073346 26339779

[7] Zhang X Wei R Wang X Zhang W Li M Ni T The neutrophil-to-lymphocyte ratio is associated with all-cause and cardiovascular mortality among individuals with hypertension. Cardiovasc Diabetol. (2024) 23(1):117. 10.1186/s12933-024-02191-5 38566082 PMC10985955

[8] Ustaalioglu İ Aydoğdu Umaç G. Neutrophil/lymphocyte ratio as a predictor of mortality among aortic dissection patients in the emergency department. Ulus Travma Acil Cerrahi Derg. (2024) 30(9):644–9. 10.14744/tjtes.2024.78241 39222496 PMC11622725

[9] Dönmez S Erdem AB Şener A Çelik GK Özdemir S Tamer S. Evaluation of descriptive performances of platelet indices, neutrophil/lymphocyte ratio, and platelet/lymphocyte ratio in aortic dissections. Rev Assoc Med Bras (1992). (2023) 69(4):e20221185. 10.1590/1806-9282.20221185 37098930 PMC10176644

[10] Chung BR Huang YT Lai PC. Can preoperative neutrophil-to-lymphocyte ratio predict in-hospital mortality in postoperative patients with Stanford type A aortic dissection? Evidence-based appraisal by meta-analysis and GRADE. Tzu Chi Med J. (2021) 33(4):388–94. 10.4103/tcmj.tcmj_249_20 34760636 PMC8532590

[11] Zhang H Guo J Zhang Q Yuan N Chen Q Guo Z The potential value of the neutrophil to lymphocyte ratio for early differential diagnosis and prognosis assessment in patients with aortic dissection. Clin Biochem. (2021) 97:41–7. 10.1016/j.clinbiochem.2021.08.002 34391696

[12] Wells GA Shea B O'Connell J. The Newcastle-Ottawa scale (NOS) for assessing the quality of non-randomised studies in meta-analyses. Ottawa Health Res Instit Web Site. (2014):7.

[13] Higgins JP Thompson SG Deeks JJ Altman DG. Measuring inconsistency in meta-analyses. Br Med J. (2003) 327(7414):557–60. 10.1136/bmj.327.7414.557 12958120 PMC192859

[14] Zhang Y Chen T Chen Q Min H Nan J Guo Z. Development and evaluation of an early death risk prediction model after acute type A aortic dissection. Ann Transl Med. (2021) 9(18):1442. 10.21037/atm-21-4063 34733994 PMC8506734

[15] Yang F Liu J Chen L Fan R Zeng H Geng Q Impact of lymphocyte-related blood parameters on short- and long-term outcomes of patients undergoing thoracic endovascular aortic repair. Angiology. (2021) 72(10):953–60. 10.1177/00033197211012514 33955277

[16] Zhao HL Tang ZW Diao YF Xu XF Qian SC Li HY Inflammatory profiles define phenotypes with clinical relevance in acute type A aortic dissection. J Cardiovasc Transl Res. (2023) 16(6):1383–91. 10.1007/s12265-023-10436-z 37713048

[17] Bedel C Selvi F. Association of platelet to lymphocyte and neutrophil to lymphocyte ratios with in-hospital mortality in patients with type A acute aortic dissection. Braz J Cardiovasc Surg. (2019) 34(6):694–8. 10.21470/1678-9741-2018-0343 31545575 PMC6894039

[18] Erdolu B As AK. C-Reactive protein and neutrophil to lymphocyte ratio values in predicting inhospital death in patients with Stanford type A acute aortic dissection. Heart Surg Forum. (2020) 23(4):E488–e92. 10.1532/hsf.3055 32726205

[19] Kalkan ME Kalkan AK Gündeş A Yanartaş M Oztürk S Gurbuz AS Neutrophil to lymphocyte ratio: a novel marker for predicting hospital mortality of patients with acute type A aortic dissection. Perfusion. (2017) 32(4):321–7. 10.1177/0267659115590625 26467992

[20] Lafçi G Ciçek ÖF Uzun HA Yalçinkaya A Diken A Turak O Relationship of admission neutrophil-to-lymphocyte ratio with in-hospital mortality in patients with acute type I aortic dissection. Turk J Med Sci. (2014) 44(2):186–92. 10.3906/sag-1301-136 25536722

[21] Onuk T Güngör B Karataş B Ipek G Akyüz S Ozcan KS Increased neutrophil to lymphocyte ratio is associated with in-hospital mortality in patients with aortic dissection. Clin Lab. (2015) 61(9):1275–82. 10.7754/Clin.Lab.2015.150216 26554247

[22] Oz K Iyigun T Karaman Z Çelik Ö Akbay E Akınc O Prognostic value of neutrophil to lymphocyte ratio and risk factors for mortality in patients with Stanford type A aortic dissection. Heart Surg Forum. (2017) 20(3):E119–e23. 10.1532/hsf.1736 28671869

[23] Zhu H Zhang L Liang T Li Y Zhou J Jing Z. Elevated preoperative neutrophil-to-lymphocyte ratio predicts early adverse outcomes in uncomplicated type B aortic dissection undergoing TEVAR. BMC Cardiovasc Disord. (2021) 21(1):95. 10.1186/s12872-021-01904-y 33593284 PMC7885432

[24] Hanna AG Contreras FJ Sharaf OM Stinson GP Hess PJ. Biomarkers to predict the outcomes of surgical intervention for aortic dissection. J Cardiothorac Surg. (2025) 20(1):116. 10.1186/s13019-024-03226-4 39910669 PMC11796221

[25] Asiyiguli Tuersun YY. Research Status of inflammation-related markers and anti-inflammatory treatment in aortic dissection. ACM. (2024) 14(9):1018–24. 10.12677/acm.2024.1492561

[26] Luo F Zhou XL Li JJ Hui RT. Inflammatory response is associated with aortic dissection. Ageing Res Rev. (2009) 8(1):31–5. 10.1016/j.arr.2008.08.001 18789403

[27] Taurino M Aloisi F Del Porto F Nespola M Dezi T Pranteda C Neutrophil-to-Lymphocyte ratio could predict outcome in patients presenting with acute limb ischemia. J Clin Med. (2021) 10(19):5–6. 10.3390/jcm10194343 PMC8509804 34640361

[28] Sirignano P Romano E Colonna G Del Porto F Margheritini C Pranteda C Neutrophil-to-Lymphocyte ratio as potential marker of outcome in popliteal artery aneurysm repair. Biomedicines. (2025) 13(3):7–9. 10.3390/biomedicines13030651 PMC11940490 40149627

[29] Yılmaz E Ak R Doğanay F. Usefulness of the neutrophil-to-lymphocyte ratio in predicting the severity of COVID-19 patients: a retrospective cohort study. Sao Paulo Med J. (2022) 140(1):81–6. 10.1590/1516-3180.2021.0298.r1.27052021 34346985 PMC9623832

[30] Xu Y Fang H Qiu Z Cheng X. Prognostic role of neutrophil-to-lymphocyte ratio in aortic disease: a meta-analysis of observational studies. J Cardiothorac Surg. (2020) 15(1):215. 10.1186/s13019-020-01263-3 32778122 PMC7419193

[31] Kılıç M Ak R Alışkan H. The utility of hemoglobin, albumin, lymphocyte and platelet (HALP) score in predicting mortality among COVID-19 patients: a preliminary study. Signa Vitae. (2023) 19(1):143–7. 10.22514/sv.2022.080

[32] Balta S Alemdar R Yildirim AO Erdogan S Ozturk C Celik T. The relationship between neutrophil-lymphocyte ratio and acute aortic dissection. Perfusion. (2017) 32(4):336–7. 10.1177/0267659116657865 28415956

