# Supplementary material for: Prognostic value of neutrophil to lymphocyte ratio in patients with aortic dissection: a systematic review and meta-analysis
Source: Front Cardiovasc Med. 2026 Jan 8;12:1631314. doi: 10.3389/fcvm.2025.1631314 (PMC12823981; doi:10.3389/fcvm.2025.1631314)
Supplement: Supplementary file 1 [file Datasheet1.pdf]

**Supplementary tables**

**Supplementary table S1** Detailed search strategy in four databases

| Database | Search strategy |
|---|---|
| Pubmed | #1 (Aortic Dissecting Aneurysm) OR (Aortic Dissecting Aneurysms) OR (Aortic Dissections) OR (Dissecting Aneurysm) OR (Dissecting Aneurysm Aorta) OR (Dissecting Aneurysm Aortas) OR (Dissecting Aneurysms) OR (Aortic Dissection[MeSH Terms])<br>#2 ((Neutrophil) OR (Polymorphonuclear Neutrophils)) OR (Polymorphonuclear Neutrophil) OR (Polymorphonuclear Leukocyte) OR (Polymorphonuclear Leukocytes) OR (LE Cells) OR (LE Cell)OR (Neutrophil Band Cells) OR (Neutrophil Band Cell) OR (Neutrophils[MeSH Terms])<br>#3 (Lymphocyte) OR (Lymphoid Cells) OR (lymphoid cell) OR ("Lymphocytes"[Mesh])<br>#4 ratio<br>#5 #2 AND #3 AND #4<br>#6 #1 AND #5 |
| Embase | #1 ratio<br>#2 aorta AND dissection OR (dissection AND of AND aorta) OR (aortic AND dissection)<br>#3 'aorta dissection'/exp<br>#4 #2 OR #3<br>#5 'neutrophil'/exp<br>#6'granulocyte, neutrophil' OR 'leucocyte, neutrophil' OR 'leukocyte, neutrophil' OR 'neutrocyte' OR 'neutrocytes' OR 'neutrophil granulocyte' OR 'neutrophil leucocyte' OR 'neutrophil leukocyte' OR 'neutrophilic granulocyte' OR 'neutrophilic leucocyte' OR 'neutrophilic leukocyte' OR 'neutrophils' OR 'pmn granulocyte' OR 'pmn leucocyte' OR 'pmn leukocyte' OR 'pmn neutrophil' OR 'polymorphonuclear granulocyte' OR 'polymorphonuclear leucocyte' OR 'polymorphonuclear leukocyte' OR 'polymorphonuclear neutrophil' OR 'polymorphous leucocyte' OR 'polymorphous leukocyte' OR 'polynuclear leucocyte' OR 'polynuclear leukocyte' OR 'neutrophil'<br>#7 #5 OR #6 |

| | |
|---|---|
| | #8 'lymphocyte'/exp<br>#9 'blood lymphocyte' OR 'f1 lymphocyte' OR 'immune competent cell' OR 'immune lymphocyte' OR 'immune lymphoid cell' OR 'immunocyte' OR 'large lymphocyte' OR 'lymph cell' OR 'lymphocyte f1' OR 'lymphocyte kinetics' OR 'lymphocyte, immune' OR 'lymphocytes' OR 'memory lymphocyte' OR 'small lymphocyte' OR 'lymphocyte'<br>#10 #8 OR #9<br>#11 #1 AND #4 AND #7 AND #10 |
| Web of Science | #1 (Aortic Dissecting Aneurysm) OR (Aortic Dissecting Aneurysms) OR (Aortic Dissections) OR (Dissecting Aneurysm) OR (Dissecting Aneurysm Aorta) OR (Dissecting Aneurysm Aortas) OR (Dissecting Aneurysms) OR (Aortic Dissection)(Topic)<br>#2 ((Neutrophil) OR (Polymorphonuclear Neutrophils)) OR (Polymorphonuclear Neutrophil) OR (Polymorphonuclear Leukocyte) OR (Polymorphonuclear Leukocytes) OR (LE Cells) OR (LE Cell)OR (Neutrophil Band Cells) OR (Neutrophil Band Cell) OR (Neutrophils)(Topic)<br>#3 (Lymphocyte) OR (Lymphoid Cells) OR (lymphoid cell) OR (Lymphocytes)(Topic)<br>#4 ratio(Topic)<br>#5 #2 AND #3 AND #4<br>#6 #1 AND #5 |
| Chochrane | #1 MeSH descriptor: [Aortic Dissection] explode all trees<br>#2 (Dissecting Aneurysm Aortas?) OR (Aortic Dissecting Aneurysm?) OR (Dissecting Aneurysm?) OR (aorta dissection)<br>#3 #1 OR #2<br>#4 (ratio)<br>#5 MeSH descriptor: [Neutrophils] explode all trees<br>#6 (Polymorphonuclear Neutrophil?) OR (Neutrophil) OR (Polymorphonuclear Leukocyte?) OR (Neutrophil Band Cell?) OR (LE Cell?) |

#7 #5 OR #6
#8 MeSH descriptor: [Lymphocytes] explode all trees
#9 (Lymphocytes) OR (Lymphocyte) OR (Lymphoid Cell?)
#10 #8 OR #9
#11 #3 AND #4 AND #7 AND #10

**Supplementary Table S2** Quality evaluation of the eligible studies with Newcastle–Ottawa scale

| | Selection | | | | Comparability | | Measurement | | |
|---|---|---|---|---|---|---|---|---|---|
| Study | Appropriateness of case ascertainment | Representativ e-ness | Selection of controls | Ascertainment of controls | Comparability on most important factors | Comparability on other risk factors | Determination of exposure factors | Exposure factors were measured in the same way | Nonresponse rate |
| Bedel 2019 | * | * | * | * | - | - | * | * | * |
| Erdolu2020 | * | * | * | * | - | - | * | * | * |
| Lafci 2014 | * | * | * | * | * | - | * | * | * |
| Kalkan 2017 | * | * | * | * | - | - | * | * | * |

*indicates criterion met; - indicates significant of criterion not met.

**Supplementary Table S3** Quality evaluation of the eligible studies with Newcastle–Ottawa scale

| Study | Selection | | | | Comparability | | Outcome | | |
|---|---|---|---|---|---|---|---|---|---|
| | Representative-ness | Selection of non-exposed | Ascertainment of exposure | Outcome not present at start | Comparability on most important factors | Comparability on other risk factors | Assessment of outcome | Long enough follow-up (median≥48 hours) | Adequacy (completeness) of follow-up |
| Yang 2021 | * | * | * | * | * | - | * | * | * |
| Zhao 2023 | * | * | * | * | * | - | * | * | * |
| Oz 2017 | * | * | * | * | - | - | * | * | * |
| Zhang 2021 | * | * | * | * | - | - | * | * | * |
| Onuk 2015 | * | * | * | * | - | - | * | * | * |
| Ustaalioglu 2024 | * | * | * | * | * | - | * | * | * |
| Zhang 2021 | * | * | * | * | - | - | * | * | * |

*indicates criterion met; - indicates significant of criterion not met.
